# Cav3.1 overexpression is associated with negative characteristics and prognosis in non-small cell lung cancer

**DOI:** 10.18632/oncotarget.24194

**Published:** 2018-01-12

**Authors:** Aleksi Suo, Allison Childers, Adrijana D’Silva, Lars F. Petersen, Shannon Otsuka, Michelle Dean, Haocheng Li, Emeka K. Enwere, Brant Pohorelic, Alexander Klimowicz, Ivana A. Souza, Jawed Hamid, Gerald W. Zamponi, DGwyn Bebb

**Affiliations:** ^1^ Department of Oncology, Tom Baker Cancer Centre, Cumming School of Medicine, University of Calgary, Calgary, AB, Canada; ^2^ Translational Laboratories, Tom Baker Cancer Centre, University of Calgary, Calgary, AB, Canada; ^3^ Functional Tissue Imaging Unit, Translational Laboratory, Tom Baker Cancer Centre, Calgary, AB, Canada; ^4^ Department of Community Health Sciences, University of Calgary, Calgary, AB, Canada; ^5^ Department of Physiology and Pharmacology, Hotchkiss Brain Institute and Alberta Children’s Hospital Research Institute, Cumming School of Medicine, University of Calgary, Calgary, AB, Canada

**Keywords:** T-type VGCC, Cav3.1, NSCLC

## Abstract

**Introduction:**

Voltage-gated calcium channels (VGCC) have been found to be differentially expressed in several different tumor types, but their role in tumor growth, malignant invasion, metastases and impact on clinical outcomes has not been clarified.

**Materials and Methods:**

From a cohort database of 193 patients with early-stage NSCLC, 163 formalin-fixed paraffin-embedded specimens were available for analysis to construct tissue microarrays. Cav3.1 protein expression was detected using fluorescence immunohistochemistry, and quantified using automated image acquisition and analysis.

**Results:**

Among the cohort of 193 NSCLC patients, adenocarcinoma (53.9%) and squamous cell carcinoma (SCC) (30.1%) were the most common histologies. There was no difference between SCC and non-SCC subtypes in overall survival (OS) or relapse-free survival (RFS); 74.2 vs 90.1 months (*p* = 0.543) and 48.8 vs 52.6 months (*p* = 0.766), respectively. T-type VGCC 3.1 (Cav3.1) overexpression was assessed by tissue microarray immunohistochemistry analysis from 163 available patient samples. Eighteen (11.0%) NSCLC primaries were found to have Cav3.1 overexpression levels, and were significantly associated with SCC histology (*p* < 0.001), larger tumor size (*p* < 0.001) and later stage disease at diagnosis (*p* = 0.019). Median OS was 48.6 vs 106.7 months for Cav3.1 overexpressing and non-overexpressing patients, respectively (*p* = 0.032). Regression analysis revealed a significantly negative effect for Cav3.1 overexpression on RFS (Hazard ratio [HR] = 2.02, *p* = 0.048).

**Conclusions:**

Cav3.1 overexpression is a potential biomarker for poorer patient outcomes. These results bring supportive evidence for calcium channels inducing an aggressive phenotype in NSCLC and potentially may serve as a therapeutic target in overexpressing tumors.

## INTRODUCTION

In 2017, an estimated 28,600 Canadians will be diagnosed with lung cancer, representing 14% of new cancer diagnoses [1]. Unfortunately, lung cancer carries a poor prognosis and is the number one cause of cancer-related mortality in Canada for both men and women, with 20,800 projected deaths in Canada for 2016.

Non-small cell lung cancer (NSCLC) histologies were previously treated the same regardless of histological subtype [2]. Outcomes were similar between squamous and non-squamous subtypes and varied little between the choice of platinum-doublet used [2, 3]. Differentiating NSCLC subtypes became clinically important with the introduction of pemetrexed and bevacizumab in the treatment of NSCLC [4–6]. Further identification of driver mutations such as EGFR and EML4-ALK altered the treatment landscape for adenocarcinoma subtypes [7]. However, there remains a gap in the understanding of cellular mechanisms regulating smoking-associated NSCLC, particularly squamous cell carcinoma (SCC).

Ion channels and pumps are increasingly being recognized to play a role in cancer cell migration, adhesion, cell-cycle control, tumor invasion and metastases [8]. Sodium, potassium, chloride and calcium channels have all been found to be differentially expressed in several different tumor types, but their role, if any, in tumor growth, malignant invasion and metastases has not been clarified [8, 9].

Calcium is a ubiquitous second messenger that plays a fundamental role in cellular migration, cell cycle control and apoptosis. A role for calcium channels in tumor proliferation has been reported in cell lines and tumors such as breast, brain, colorectal, gastric, hepatic, prostate, leukemia, retinoblastoma and pheochromocytoma [10–12], and silencing of calcium channel expression has been shown to reduce proliferation [13–16]. Bioinformatics gene expression analysis have revealed the upregulation of voltage-gated calcium channels (VGCC) in many types of cancers, including lung cancer [17]. Consequences of altered calcium signaling in cancer cells has been thought to contribute to tumor progression, suggesting that calcium channels and pumps may serve as additional therapeutic targets for overexpressing cancer subtypes [18, 19].

In this study, we analyzed T-type VGCC 3.1 (Cav3.1) protein expression by tissue microarray immunohistochemistry analysis and correlated it to clinical characteristics and patient outcomes in a cohort of early-stage NSCLC patients.

## RESULTS

### Cohort characteristics

One hundred and ninety-three patients with early stage (I-III) resectable NSCLC were identified from the Glans-Look Lung Cancer Database (GLD) from 2003–2006 (Table 1); 118 with stage I, 55 with stage II, 19 with stage III, and one patient with pulmonary carcinoid and a resected solitary liver metastasis. The average age was 64-years and most were current or former smokers (87.6%). Adenocarcinoma (53.9%) and SCC (30.1%) were the predominant histology subtypes. Average primary lung tumor size was 3.5 cm. Seventy-seven patients (39.9%) received adjuvant chemotherapy at some point in their treatment. 32.1% of patients eventually relapsed with metastatic disease.

**Table 1:** Characteristics of stage I–III NSCLC patients from the GLD diagnosed between 2003–2006 (*n* = 193)

Factors	*n* (%)
Age in years, median (min-max)	64.0 (30–84)
Gender	
Female	101 (52.3)
Male	92 (47.7)
Tobacco use	
Never	24 (12.4)
Current or former	169 (87.6)
Histology	
Adenocarcinoma	104 (53.9)
Squamous^a^	58 (30.1)
Adenocarcinoma in situ	11 (5.7)
Large cell neuroendocrine	7 (3.6)
Large cell	6 (3.1)
Other^c^	7 (3.6)
Tumor size in cm, median	3.5 (0.5–15.0)
TNM staging	
Stage IA	41 (21.2)
Stage IB	77 (39.9)
Stage IIA	28 (14.5)
Stage IIB	27 (14.0)
Stage IIIA	18 (9.3)
Stage IIIB	1 (0.5)
Stage IV^b^	1 (0.5)
Metastases developed	62 (32.1)
Adjuvant chemotherapy	77 (39.9)

^a^Five patients had adenosquamous subtype.

^b^One patient with pulmonary carcinoid and solitary liver metastases treated with surgical resection.

^c^Includes carcinoid subtypes, giant cell, sarcomatid and adenocarcinoma with neuroendocrine features.

### Cohort outcomes: squamous and non-squamous

**Figure 1:**
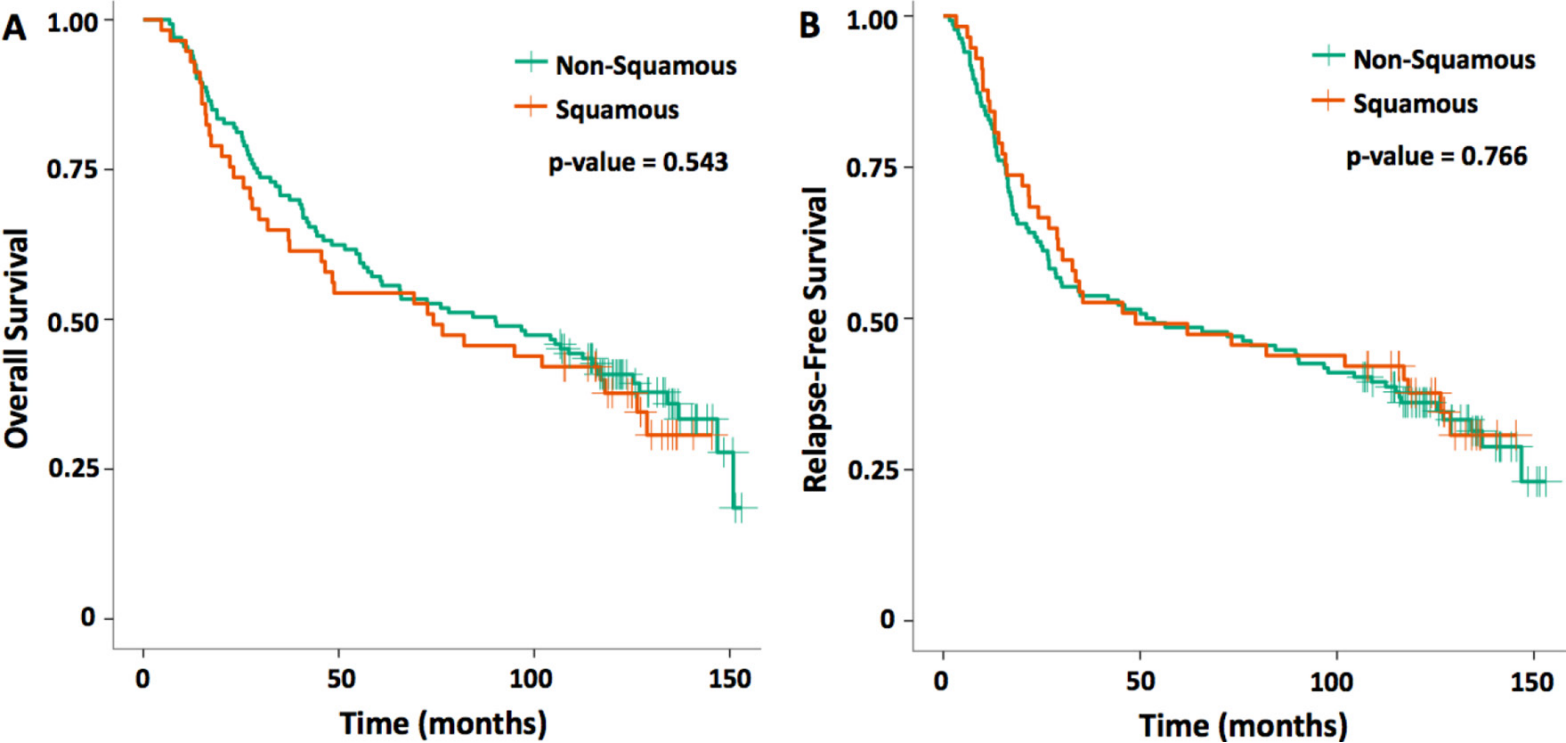
Kaplan-Meier curves for squamous versus non-squamous cell histology (**A**) OS and (**B**) RFS for the early-stage NSCLC. One hundred and ninety-three patients were included in this cohort analysis from 2003–2006.

Kaplan-Meier overall survival (OS) and relapse-free survival (RFS) curves for SCC vs non-SCC NSCLC patients from the GLD are demonstrated in Figure 1. Estimates between SCC and non-SCC patients found no statistically significant differences in median OS at 74.2 and 90.1 months, respectively (*p* = 0.543). Median RFS was also similar at 48.8 for SCC and 52.6 months for non-SCC patients (*p* = 0.766).

### T-type voltage gated calcium channel expression and clinical-pathological characteristics

**Figure 2:**
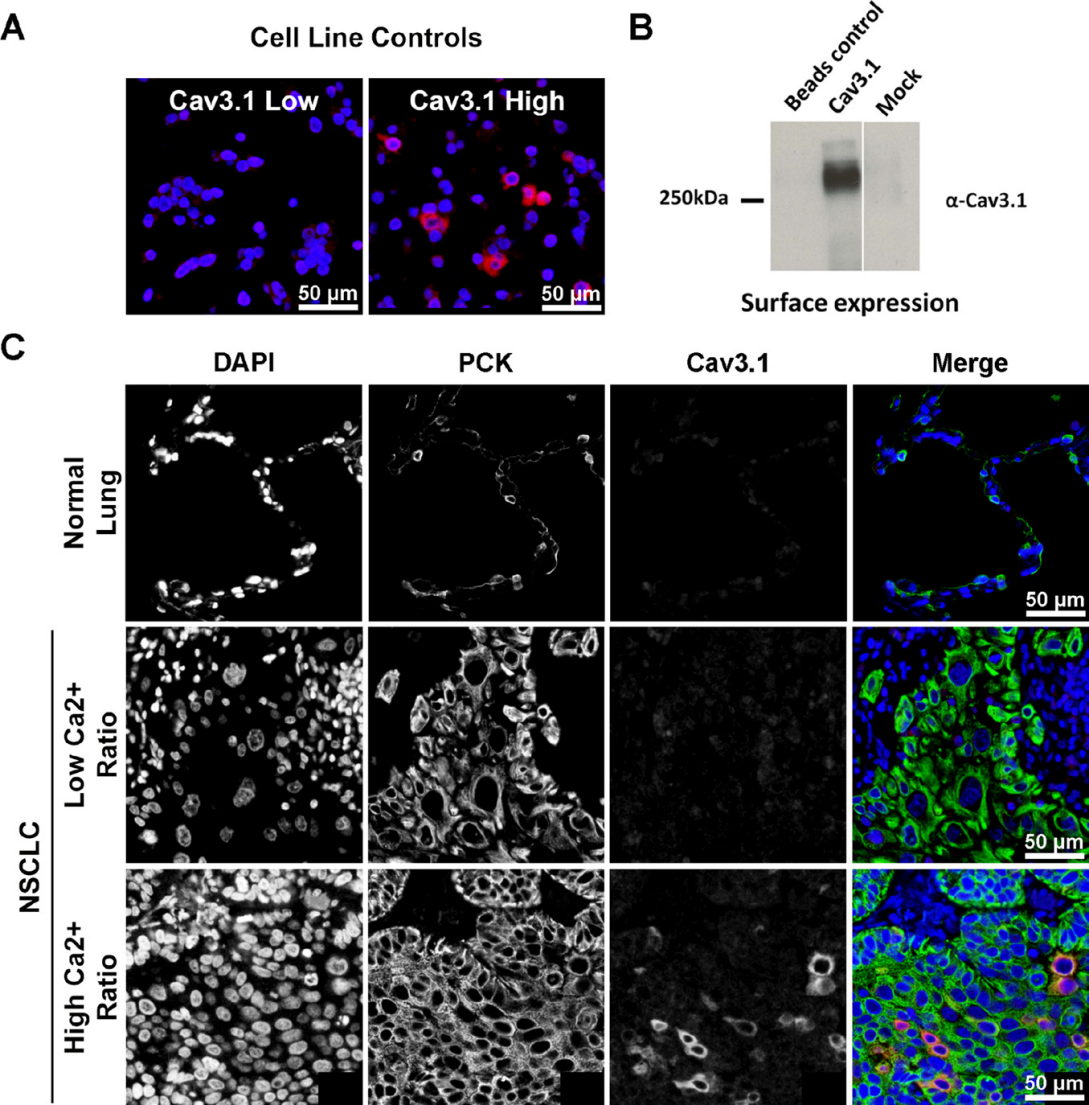
Immunofluorescent staining for Cav3.1 For each panel, (**A**) control staining of non-transfected tsA-201 cells, and cells transiently transfected with Cav3.1 cDNA. (**B**) Surface biotinylation of Cav3.1 channels transfected in tsA-201 cells. Beads control consists of cells that were not treated with biotin. Mock represents cells that were mock transfected. (**C**) Examples of Cav3.1 protein staining in benign lung tissue (row 1), NSCLC with low expression (row 2), and overexpression (row 3). The merge panels are pseudo-colored blue for DAPI, green for pan-cytokeratin, and red for Cav3.1. Photos are exposure adjusted for visual illustration of signal localization. DAPI = 4’,6-diamidino-2-phenylindole; PCK = pan-cytokeratin.

From the GLD cohort of 193 patients with early-stage NSCLC, 163 formalin-fixed paraffin-embedded specimens were available for analysis to construct tissue microarrays. Tissue samples were analyzed for T-type VGCC 3.1 (Cav3.1) expression (Figure 2). Eighteen (11.0%) of 163 NSCLC primary tumors were found to have Cav3.1 overexpression (Table 2). Fisher’s exact testing found statistically significant differences in characteristics between Cav3.1 overexpressing and low expressing tumors. Lung cancers with overexpression of Cav3.1 were more likely to be of squamous cell histology (*p* < 0.001), larger tumor size (*p* < 0.001) and later stage at diagnosis (*p* = 0.019), than tumors with less Cav3.1 expression. Of the SCC samples (including five adenosquamous histology), 31.4% were found to be overexpressing Cav3.1, and 88.9% of Cav3.1 overexpressing tumors were SCC. Median tumor size for Cav3.1-overexpressing tumors were 6.00 cm compared to 3.35 cm for non-overexpressing tumors. 22.2% of patients with Cav3.1-overexpression had stage III disease at presentation, compared to only 7.6% of patients with non-overexpression. Age, gender, current or former smokers, later development of metastases or treatment with adjuvant chemotherapy had no statistical relationship to Cav3.1 expression.

**Table 2:** Characteristics associated with Cav3.1+ (*n* = 18) and Cav3.1– (*n* = 145) expression on primary NSCLC tumor samples

Factors	Cav3.1+ *n* (%)	Cav3.1– *n* (%)	*p*-value
Age in years, median	66	64	0.347
Gender			0.218
Female	7 (38.9)	80 (55.2)	
Male	11 (61.1)	65 (44.8)	
Tobacco use			0.135
Never	0 (0.0)	22 (15.2)	
Current or former	18(100.0)	123(84.8)	
Histology			**< 0.001**
Squamous^a^	16 (88.9)	35 (24.1)	
Non-squamous	2 (11.1)	110 (75.9)	
Tumor size in cm, median	6.00	3.35	**0.001**
TNM Staging			**0.019**
Stage IA	2 (11.1)	33 (22.8)	
Stage IB	3 (16.7)	64 (44.1)	
Stage IIA	5 (27.8)	18 (12.4)	
Stage IIB	4 (22.2)	19 (13.1)	
Stage IIIA	4 (22.2)	10 (6.9)	
Stage IIIB	0 (0.0)	1 (0.7)	
Metastases developed	8 (44.4)	41 (28.3)	0.178
Adjuvant chemotherapy	5 (27.8)	61 (42.1)	0.313

All stage I-III NSCLC patients with available Cav3.1 expression analysis from 2003-2006 in the GLD were included.

^a^Includes all squamous and adenosquamous cell histologies

### T-type voltage gated calcium channel expression and associated patient outcomes

**Figure 3:**
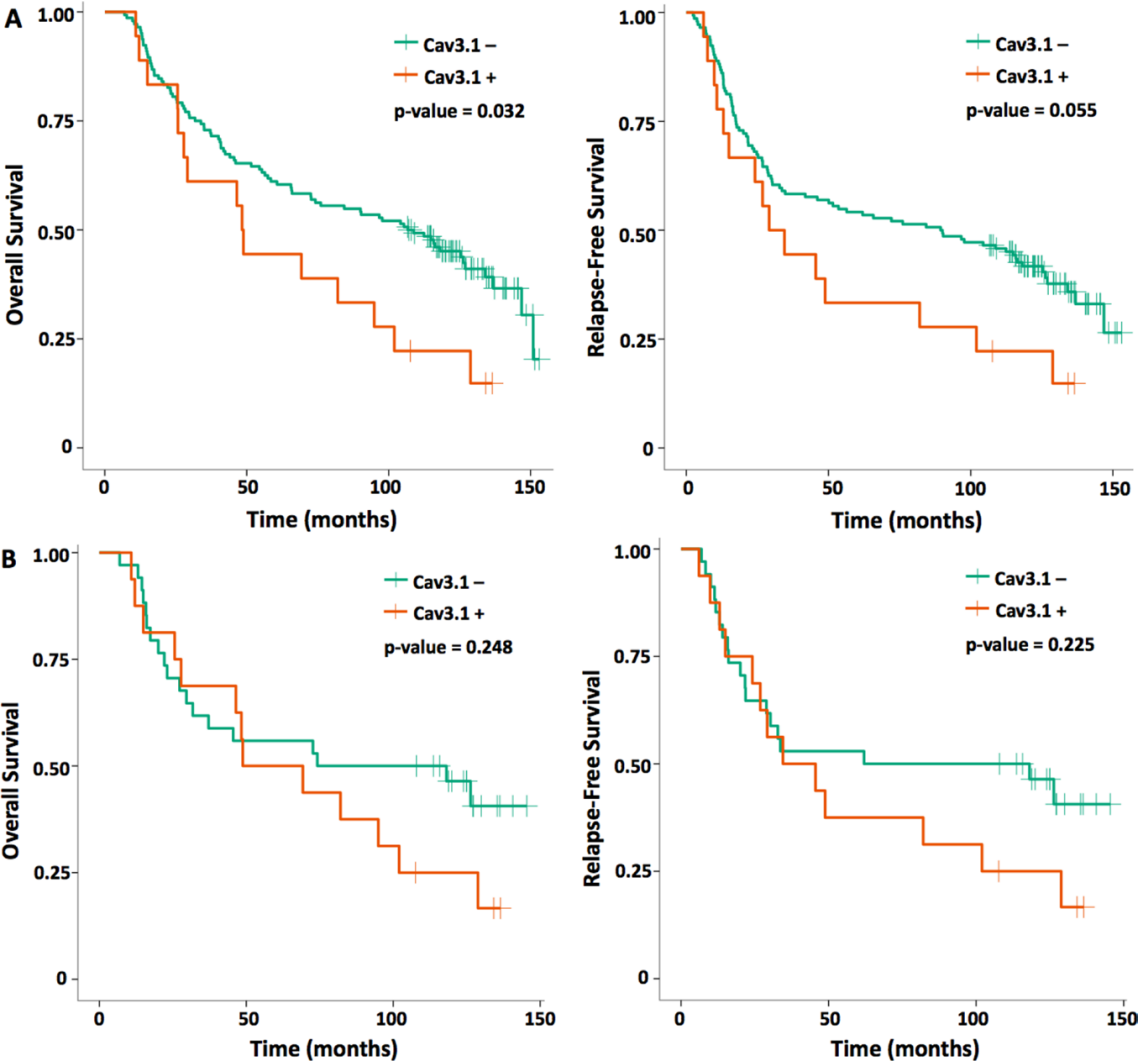
OS and RFS Kaplan-Meier curves for (**A**) all NSCLC patients and (**B**) SCC histology only based on voltage-gated calcium channel (Cav3.1) expression.

Kaplan-Meier survival estimates and comparisons on log rank testing for 163 NSCLC patients was also analyzed based on Cav3.1 expression (Figure 3 and Table 3). Patients with overexpressing tumors had statistically significantly worse outcomes compared to the non-overexpressing patients. Median OS for Cav3.1 overexpressing patients was 48.6 months compared to 106.7 months for non-overexpressing patients (*p* = 0.032). Median RFS was 31.9 vs 89.8 months for the Cav3.1 overexpressing and non-overexpressing patients, respectively, and was trending toward statistical significance (*p* = 0.055). Subgroup analysis of SCC patients revealed a numerical reduction in OS by 37 months and RFS by 50 months for Cav3.1+ tumors; however, these differences were not statistically significant, possibly owing to the small sample size (see Supplementary Tables 1–3 for additional SCC subgroup analyses).

**Table 3:** Median survival outcomes in months for patients with calcium channel overexpressing and non-overexpressing NSCLC

	Cav3.1+	Cav3.1–	*p*-value
All NSCLC (*n* ***=*** 163)			
OS	48.6	106.7	**0.032**
RFS	31.9	89.8	0.055
Squamous^a^ only (*n* ***=*** 51)			
OS	59.1	96.2	0.248
RFS	40.1	90.1	0.225

^a^Squamous and adenosquamous histologies included.

After adjusting for SCC histology, gender, smoking, adjuvant therapy and tumor size in the multivariable Cox proportional-hazards model (Table 4), Cav3.1 overexpression maintained a statistically significant negative effect on RFS (Hazard ratio [HR] = 2.02, *p* = 0.048). A trend towards poorer OS (HR = 1.88, *p* = 0.075) was also noted for Cav3.1 overexpression, although this did not uphold statistical significance. However, none of the other variables of SCC histology, gender, smoking, adjuvant therapy and tumor size had any effect on OS or RFS after regression analysis.

**Table 4:** Multivariable Cox proportional-hazards regression models for OS and RFS in NSCLC

Variable	Hazard ratio	95% CI^a^	*p*-value
OS			
Squamous	0.91	0.53–1.56	0.724
Sex (male)	1.07	0.70–1.62	0.757
Cav3.1 expression	1.88	0.94–3.76	0.075
Smoking	0.73	0.38–1.40	0.347
Adjuvant treatment	1.12	0.73–1.72	0.602
Tumor size	1.01	0.92–1.11	0.877
RFS			
Squamous	0.71	0.41–1.23	0.225
Sex (male)	1.18	0.78–1.77	0.440
Cav3.1 expression	**2.02**	1.01–4.05	**0.048**
Smoking	0.82	0.44–1.53	0.530
Adjuvant treatment	1.12	0.74–1.69	0.587
Tumor size	1.03	0.94–1.13	0.550

^a^Confidence Interval

## DISCUSSION

Here, we demonstrated for the first time that a subset of tumors from an early-stage NSCLC cohort overexpress the T-type VGCC 3.1 (Cav3.1) by tissue microarray immunohistochemistry analysis. In addition, we demonstrated that Cav3.1 overexpression is significantly associated with squamous cell histology, larger tumor size and later stage disease at diagnosis. Furthermore, Cav3.1 overexpression was associated with a poorer OS, and was found to be an independent predictor for shorter RFS in the regression analysis.

Ion channels and pumps are increasingly being recognized to play a role in carcinogenesis. Studies have reported dysregulation of potassium channel expression in breast, colon, prostate and brain tumors [8]. Voltage-gated sodium channels have been found to be differentially expressed in breast, colon, cervix, prostate, brain and NSCLC. Sodium-proton exchangers have been demonstrated to play a role in malignant invasion and metastasis, and dysregulation of chloride channels in cancers have also been described [8].

Calcium is a ubiquitous second messenger that contributes to many fundamental cellular processes including contraction, motility, transmitter release, exocytosis and endocytosis. It also plays essential roles in cell cycle control, proliferation, growth, migration and apoptosis which are necessary characteristics for malignant cells to adopt. These hallmark traits of cancer are controlled by a myriad of intracellular signaling networks, many of which require calcium to regulate kinases, phosphatases, cyclases, esterases and ion channels [17, 20]. The timing, extent, location and duration of the intracellular calcium oscillations determine the impact it has on cellular function.

Cytoplasmic calcium levels are regulated through calcium channels on the endoplasmic reticulum (ER), mitochondrial surface and cell membrane [21]. Inositol-1,4,5-trisphosphate (IP_3_) signaling releases calcium stores in the ER with subsequent activation of store operated calcium channels on the plasma membrane [21–23]. Additional mechanisms for regulating intracellular calcium levels are through VGCC on the plasma membrane, which are functionally expressed on many cell types and epithelial cancers. VGCCs are mainly of the L (long-lasting)- or T (transient)-type variety, although other VGCCs and ligand-mediated calcium channels exist [23, 24]. A sustained elevation of intracellular calcium concentrations induces dephosphorylation and activation of the NFAT transcription factors via calcineurin. Members of the NFAT transcription family have been shown to induce expression of genes involved in cell survival, growth, proliferation, angiogenesis, invasive migration and the tumor microenvironment [23, 25].

Various studies have investigated calcium channel expression levels in cell lines and tissue specimens [10-16]. Transient receptor potential calcium channels like TRPC3 and TRPC6 have been found to be elevated in breast, ovarian, liver and stomach tumors, and silencing of calcium channel expression has been shown to reduce proliferation in a breast cancer cell line and reduce tumor formation in a xenograft mouse model [13, 14]. T-type VGCC overexpression has been shown in many cancer cell lines [16, 17, 26, 27]. Several gene silencing experiments of these channels have demonstrated reduced proliferation [15, 16], whereas upregulation of T-type VGCC increases cell proliferation *in vitro* [28, 29]. Hypoxia has also been shown to upregulate Cav3.1 and Cav3.2 expression and cell proliferation in melanoma cells, suggesting that relative hypoxia in the tumor microenvironment may induce tumor growth and angiogenesis [29–31]. Glioma-specific Cav3.1 gene regulation and splice variants have also been documented, supporting a possible contribution to tumor pathogenesis [32]. These investigative results prompted our current study characterizing VGCC in a NSCLC cohort, and are consistent with our results where Cav3.1 overexpression was significantly associated with larger tumors at diagnosis.

Moreover, Sakakura *et al.* found expression of inositol 1,4,5-triphosphate receptor type 3 (ITPR3) to be overexpressed in gastric cancer taken from peritoneal metastases compared to gastric primary cell lines [33]. ITPR3 regulates the mobilization of intracellular calcium stores, and the authors conclude that IP3R3 signaling may contribute to peritoneal dissemination of gastric cancer. Noteworthy, we found calcium channel overexpression to be associated with later stage disease at diagnosis, also suggesting that increased calcium signaling may lead to earlier locoregional lymph node spread of lung cancer. This could also explain the poorer survival outcomes in patients with Cav3.1 overexpression from our study. A more recent investigation examining an N-type VGCC in NSCLC found that overexpression levels of this type of calcium channel was also linked to larger tumors, later stage disease and lower OS [34]. Accumulating evidence suggests that VGCCs and calcium-regulated signaling do play a role in the hallmarks of cancer such as proliferation, motility and invasion [24].

A bioinformatics meta-analysis on public microarray datasets found mRNA upregulation of different VGCC subtypes across various cancers [17]. Cav3.1 mRNA was upregulated in sarcoma, colorectal, uterus, lung, prostate and breast. Interestingly, Cav3.1 was in the top 1% and 2% of overexpressed genes in sarcoma and prostate, respectively, and was increased almost 2-fold in adenocarcinoma of the lung. In contrast, Cav3.1 protein expression in our cohort was strongly associated with SCC rather than other subtypes of NSCLC despite being found in only 31% of SCC patients. Cav3.1 has been discovered in other SCC cells, although this was in laryngeal head and neck cancer rather than lung [35]. Zhou *et al.* investigated an N-type VGCC (Cav2.2) in NSCLC, where increased Cav2.2 expression was revealed in 51% of lung adenocarcinomas, and was even higher in squamous (63%) and adenosquamous (89%) subtypes [34].

The prospect of T-type VGCCs as a potential therapeutic target has been suggested [36]. One study demonstrated the knockdown of Cav3.1 reduced cell growth and induce apoptosis in colon cancer cells [37]. The T-type calcium channel blocker, mibefradil, has Cav3.1 antagonist activity and was initially developed as an antihypertensive agent, but was withdrawn from the market for cross reactions with other medications and toxicity [38]. Nonetheless, mibefradil has been shown to reduce cell motility and invasion in a fibrosarcoma cell line [26]. Additional small molecule T-type calcium channel inhibitors are being studied and have shown anticancer activity *in vitro* [24].

If a phenotype for aggressive tumor growth and metastasis can be initiated by increasing NFAT-regulated gene expression, cancer could achieve calcium dysregulation through a multitude of avenues with the same result. Further studies should focus on the acquisition of calcium signaling and NFAT-regulated gene expression with the comparison of primary to metastatic sites. These studies could better characterize calcium’s role in the development of metastases, a crucial event related to poorer patient outcomes. Furthermore, T-type channel currents are rarely observed in Cav3.1 expressing NSCLC cell lines (Supplementary Figures 1–3), and these channels may engage in other possible mechanisms of signaling independent of T-type current activity in the plasma membrane. Indeed, T-type calcium channel expression has been reported in the nucleus of certain types of cells [39]. Whether such a mechanism could contribute to the pathophysiological role of these channels in cancer cells will be a focus of future investigation.

Bioinformatics data has provided clues as to which ion channel genes may have functional roles in cancer, and additional studies identifying overexpressed proteins in cancer cell lines corroborate their significance. Nevertheless, the linking of biologic tumor characteristics to real-world clinical outcomes is essential in separating mutational robustness from gene expression noise.

Here we demonstrate Cav3.1 overexpression is associated with larger tumors, SCC histology, later stage disease at diagnosis and lower OS in patients with NSCLC, and is a potential biomarker for poorer patient outcomes.

This study is limited by its retrospective nature and relatively low numbers of SCC subtypes and Cav3.1 overexpressing samples. However, this study brings supportive evidence for calcium channels inducing an aggressive phenotype in NSCLC and their potential as a therapeutic target in overexpressing tumors.

## MATERIALS AND METHODS

### Case selection and clinical data collection

This study was approved by the University of Calgary Conjoint Faculties Research Ethics Board, in accordance with the Tri-Council Policy Statement on Research with Human Subjects. Clinical data were collected retrospectively through chart review of NSCLC patients diagnosed at the Tom Baker Cancer Centre (TBCC) from 2003 to 2006 and entered into the Glans-Look Lung Cancer Database (GLD). All patients diagnosed during this period as identified by the provincially legislated Alberta Cancer Registry were included. Relevant data were obtained from physician progress notes, pathology reports, diagnostic imaging reports and laboratory results. Demographic details included age at diagnosis, gender and smoking status. Clinical variables included stage of disease, tumor histology, treatment modalities, and outcome data. Staging was performed according to the American Joint Committee on Cancer tumor, node, metastasis system and reflected the 2009 revisions for NSCLC staging.

### Tissue microarray generation

All available archived formalin-fixed paraffin-embedded (FFPE) tumor samples from stage I-III NSCLC patients included in the clinical database were retrieved from Calgary Laboratory Services. Hematoxylin and eosin-stained slides were reviewed by a pathologist to confirm diagnosis, and those deemed to be of sufficient quality were selected and marked for sampling and inclusion into the tissue microarray (TMA). Representative cores (0.6 mm) from each specimen were assembled in triplicate (when adequate material was available) into each TMA (25–45 specimens per TMA) using a Beecher Manual Tissue Microarrayer (Beecher Instruments Inc., Sun Prairie, WI, USA). Normal lung tissue specimens and Hela cells were also included as controls.

### Protein expression controls

Human embryonic kidney tsA-201 cells were grown, maintained and transiently transfected with human Cav3.1 and GFP cDNA constructs as described previously using the calcium phosphate method [40, 41]. Following expansion to ten million cells per condition, cells were harvested, formalin-fixed, suspended in Histogel (HG-4000-012, Thermo Scientific), and paraffin-embedded as described [42]. For the biotinylated Western blot, cells were washed with HBSS and incubated on ice for 15 min to stop trafficking of proteins. Cells were then incubated with either HBSS only (beads control) or with HBSS containing 1 mg/ml EZ-Link Sulfo-NHS-SS-Biotin (Thermo Scientific) for 1 hour on ice. The reaction was quenched with 100 mM Glycine and cells were lysed in modified RIPA buffer. Biotinylated proteins were precipitated using Neutravidin beads (Thermo Scientific) and resolved by SDS-PAGE followed by Western Blot using a rabbit anti-Cav3.1 antibody (Alomone).

Representative cores (0.6 mm) from each cell block were incorporated into a testing array alongside cores from various normal and cancer FFPE tissue blocks into TMAs that served as assay controls for each stain.

### Fluorescence immunohistochemistry

TMA sections (4 μm) were deparaffinized and rehydrated as previously described [43]. Heat-induced epitope retrieval was performed using a decloaking chamber (Biocare Medical, Concord, CA, USA) by heating slides to 121°C for 1 minute, in a citrate-based (pH 6.0) target retrieval solution (S1699, DAKO, Mississauga, Canada). Immunostaining was performed on a DAKO Autostainer as previously described [44]. Generation of the Cav3.1 rabbit polyclonal antibody was described previously [45]. The antibody was diluted to 1:10,000 in Signal Stain^®^ protein block (8112L, Cell Signaling, Danvers, MA, USA) and applied at room temperature for 60 minutes along with pan-cytokeratin to identify tumor epithelia (mouse clone AE1/AE3, M3515, 1:100, DAKO). Following three washes in TBST, anti-rabbit EnVision+ (K4011, DAKO) secondary antibody containing anti-mouse Alexa 555-conjugated secondary antibody (A21424, 1:200, Thermo Scientific, Burlington, ON, Canada) was applied for 60 minutes, and visualized with TSA-Plus Cy5 signal amplification reagent for 5 minutes (Perkin Elmer, Waltham, MA, USA). After immunostaining, slides were coverslipped using ProLong Gold anti-fade mounting medium with diamidino-2-phenylindole (DAPI) (P36935, Thermo Scientific), and stored at 4°C until scanned.

### Automated image acquisition and analysis

Automated image acquisition was performed using the Aperio Scanscope^®^ FL (Aperio Inc., Vista, CA, USA) slide scanner and images were analyzed using the HistoRX AQUAnalysis^®^ program, version 2.4.4.1 (Genoptix, Carlsbad, CA, USA) [42, 46]. Briefly, seamless high-resolution images for each TMA core were acquired using filters specific for DAPI to define the nuclear compartment, Cy3 to define the cytokeratin-positive tumor cytosolic compartment, and Cy5 to define Cav3.1 expression. A tumor-specific mask was generated to distinguish NSCLC cells from surrounding stromal tissue by thresholding the pan-cytokeratin images, and a similar mask was created to identify T-type calcium channels by thresholding the Cav3.1 images. Cell line cores serving as protein expression controls used to train the algorithm were non-transfected tsA-201 cells and tsA-201 cells that were transiently transfected with human Cav3.1 cDNA. All subsequent images were processed using these optimal threshold values.

For each core, a Cav3.1-positive tumor ratio (Ratio) representing the percent of the tumor mask pixel area occupied by the pixel area of the Cav3.1-positive mask was calculated. The mean Ratio over triplicate spots, where available, was used for statistical analysis. The cut-off value for Cav3.1 overexpressing cells was anything above 0, and the pixel masking threshold was determined using specific cytoplasmic staining in the transiently transfected tsA-201 cells.

### Statistical analysis

Fisher’s exact test was used to analyze categorical data, and Wilcoxon rank-sum test was performed to study continuous variables. Overall survival (OS) and relapse-free survival (RFS) outcomes were analyzed using the Kaplan-Meier method, with the log-rank test to compare groups. Cox regressions were used to jointly evaluate the influence from Cav3.1 expression and other factors (e.g. sex, tumor size, etc.). All analyses were implemented by R v3.3.0 [47].

## SUPPLEMENTARY MATERIALS FIGURES AND TABLES


