# Supplementary material for: Cav3.1 overexpression is associated with negative characteristics and prognosis in non-small cell lung cancer
Source: Oncotarget. 2018 Jan 12;9(9):8573–83. doi: 10.18632/oncotarget.24194 (PMC5823575; doi:10.18632/oncotarget.24194)
Supplement: Supplementary file 1 [file oncotarget-09-8573-s001.pdf]

Cav3.1 overexpression is associated with negative characteristics and prognosis in non-small cell lung cancer

SUPPLEMENTARY MATERIALS

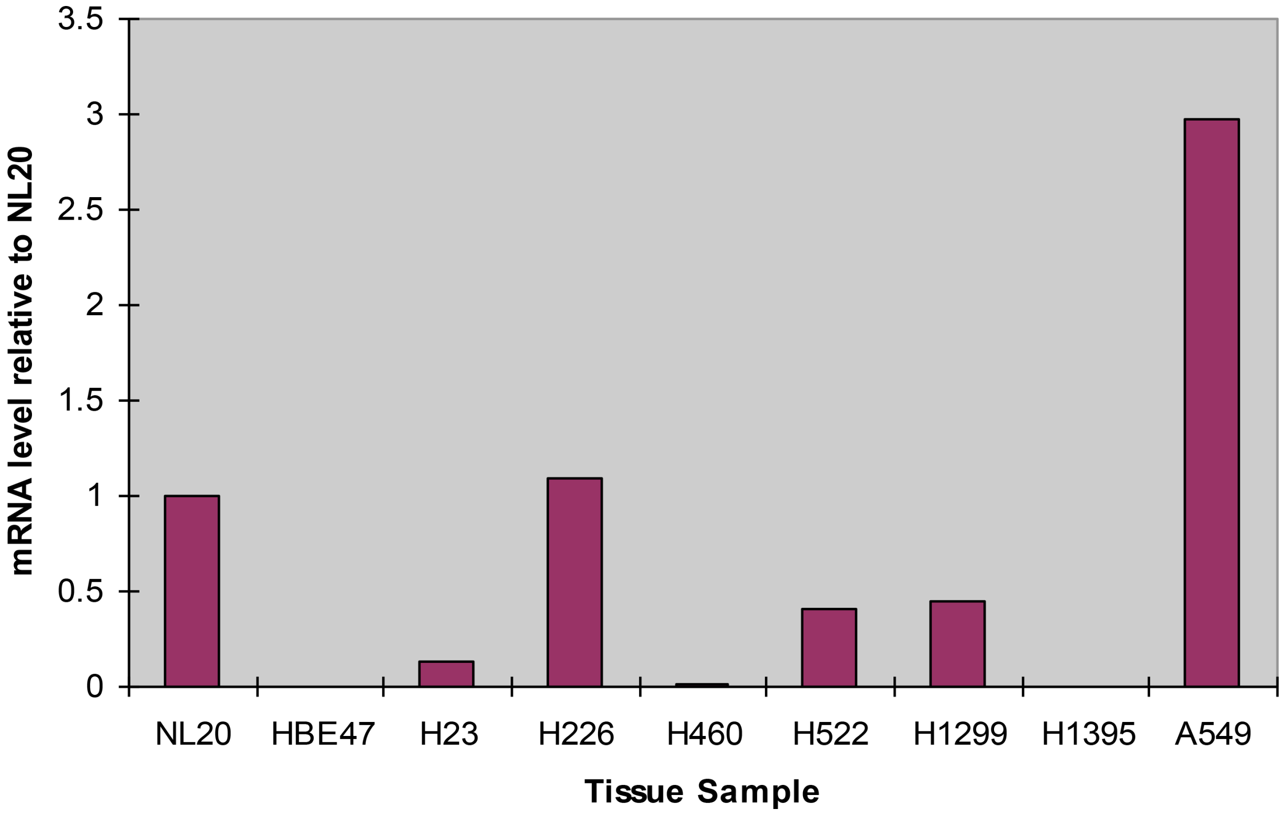

Supplementary Figure 1: CACNA1G mRNA expression levels in NSCLC cell lines.

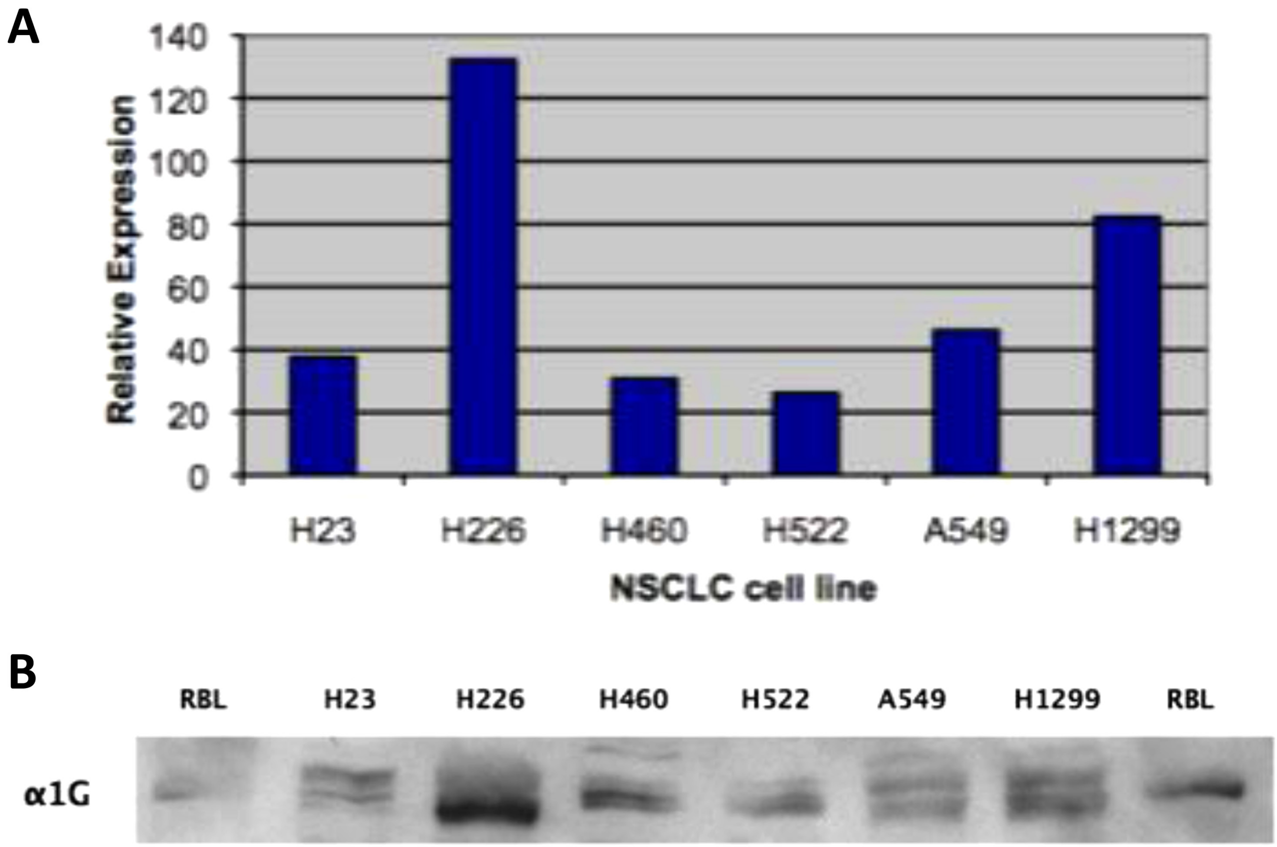

Supplementary Figure 2: Cav3.1 expression densitometry from NSCLC cell lines. (**A**) Relative protein expression levels from seven NSCLC cell lines calculated by (**B**) Western blot analysis with RBL cell line controls.

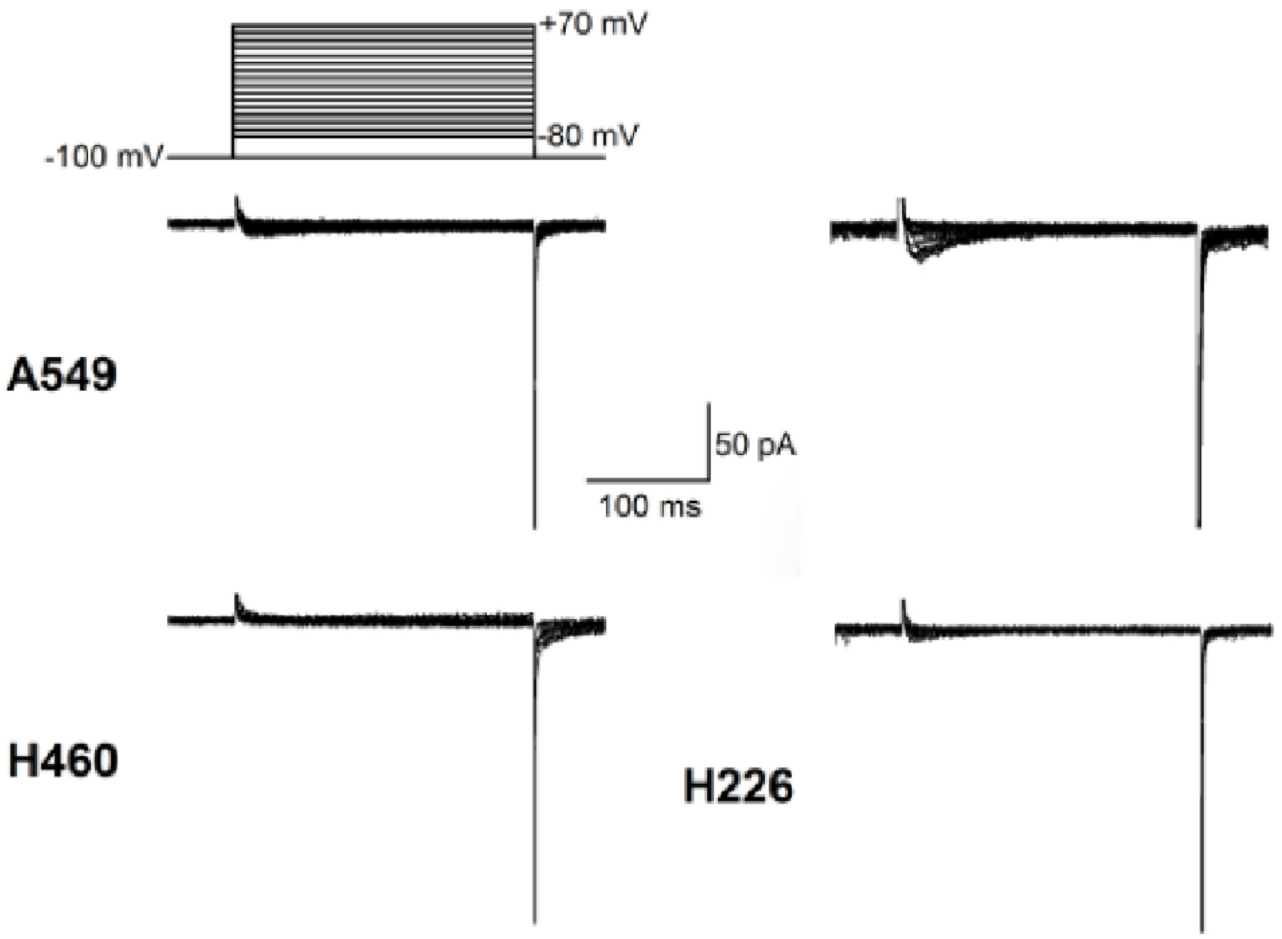

Supplementary Figure 3: Patch-clamp functional studies of A549 Cav3.1 expressing cells. Twenty-five total attempts were carried out with currents observed in only two A549 cells. External recording solution: (in mM) 10 BaCl2, 1 MgCl2, 125 CsCl, 10 Hepes, and 10 glucose (pH 7.4). Internal recording solution (in mM): 130 CsCl, 2.5 MgCl2, 10 Hepes, 5 EGTA, 3 ATP, 0.5 GTP (pH 7.4).

Supplementary Table 1: Characteristics of all SCC subroup analysis patients only

Factors	*n* (%)
**Adenosquamous**	5
**Squamous**	53
**Developed Metastases**	
**Yes**	20 (34.5)
**No**	38 (65.5)
**Median tumor size, cm**	3.9
**Previous or current smoker**	58 (100)
**Median age, years**	67
**Female sex**	19 (32.8)

Supplementary Table 2: SCC subgroup analysis of patients with available Cav3.1 data only, *N* = 51

Factors	Cav3.1+ *n* (%)	Cav3.1- *n* (%)	*p*-value
Total patients	16 (31.4)	35 (68.6)	
Age in years, median	66.5	68	0.895
Female sex	6 (37.5)	10 (28.6)	0.534
Developed metastases	7 (43.8)	11 (31.4)	0.529
Tumor size in cm, median	5.7	3.8	0.170
Received adjuvant treatment	5 (31.3)	15 (42.9)	0.543
Stage			0.308
IA	2	5	
IB	3	15	
IIA	5	4	
IIB	3	7	
IIIA	3	4	

Supplementary Table 3: Multivariable Cox proportional-hazards regression models for OS and RFS in SCC subgroup patients only

Variable	HR	95% CIa	p-value
**OS**			
**Male**	0.94	0.43-2.02	0.864
**Cav3.1**	1.72	0.81-3.68	0.160
**Received adjuvant treatment**	1.35	0.61-3.01	0.462
**Tumor size**	0.90	0.76-1.08	0.276
**PFS**			
**Male**	0.87	0.40-1.89	0.731
**Cav3.1**	1.72	0.81-3.62	0.156
**Received adjuvant treatment**	1.31	0.59-2.91	0.502
**Tumor size**	0.90	0.75-1.08	0.272

[a]Confidence Interval.
